# Calcified frontal neurocysticercosis presenting with acute psychosis in a non-endemic context: a case report

**DOI:** 10.3389/fpsyt.2026.1771798

**Published:** 2026-03-09

**Authors:** Mohammed Salah Alfahal, Tala Jalkhi

**Affiliations:** Department of Psychiatry, Rashid Hospital, Dubai Academic Health Corporation (DAHC), Dubai, United Arab Emirates

**Keywords:** acute psychosis, case report, frontal lobe, migration, neurocysticercosis, neuropsychiatry, secondary psychosis

## Abstract

**Background:**

Neurocysticercosis (NCC) is the most common parasitic infection of the central nervous system and a widely recognized cause of seizures; nonetheless, its psychiatric manifestations, while recorded, are frequently underdiagnosed, particularly in non-endemic settings. Acute psychosis secondary to NCC is rather uncommon and hard to diagnose, as it often mimics primary psychiatric illness.

**Case presentation:**

We report the case of a 23-year-old Indian male living in the United Arab Emirates who presented with two days of confusion, agitation, mutism, disorganized behavior, and persecutory delusions following severe sleep deprivation in a labor camp setting. His history included a previous similar psychotic episode with full remission and two generalized tonic–clonic seizures, the most recent occurring three weeks before admission. On arrival, he exhibited psychomotor abnormalities, intermittent restlessness, and incoherent responses, without focal neurological deficits. Laboratory investigations were unremarkable except for mild lymphocytosis and elevated creatine phosphokinase. CT Brain revealed a 3 mm calcified cortical lesion with perilesional edema in the inferior posterior left frontal lobe, consistent with calcified parenchymal NCC. EEG was normal. He was co-managed by neurology and psychiatry teams and initiated on albendazole, dexamethasone, lamotrigine, risperidone, and lorazepam. His symptoms rapidly improved, with complete resolution of psychosis within four days. Post-discharge MRI in India confirmed a solitary ring-enhancing lesion with central calcification and mild edema compatible with NCC. Upon follow-up, he remained stable with no recurrent seizures or psychiatric symptoms.

**Conclusion:**

This case demonstrates acute, reversible psychosis associated with calcified frontal-lobe neurocysticercosis, highlighting the importance of neuroimaging in first-episode or atypical psychosis, particularly in patients from endemic regions or with a history of seizures. Clinical improvement following combined antiparasitic, anti-inflammatory, antiepileptic, and antipsychotic therapy supports the value of early multidisciplinary management, while recognizing that symptom resolution likely reflects multifactorial influences. As global migration increases, clinicians in non-endemic regions should remain aware of NCC as a potential contributor to secondary psychosis.

## 1 Introduction

Neurocysticercosis (NCC), caused by the larval stage of Taenia solium, is the most common parasitic infection of the central nervous system (CNS) and a major cause of acquired epilepsy worldwide (1). It is estimated that NCC accounts for up to 30% of epilepsy cases in endemic regions, making it a significant contributor to neurological disability and socioeconomic burden (2). Globally, between 2.56 and 8.30 million individuals are estimated to have symptomatic or asymptomatic NCC, based on available epilepsy prevalence data (3). Even in high-income countries such as the United States, more than 1,800 hospitalizations annually are attributed to NCC, with healthcare expenditures exceeding those of all other neglected tropical diseases combined. The global public health impact of NCC is further underscored by its classification as a neglected tropical disease, reflecting persistent gaps in diagnosis, surveillance, and control strategies (4). While the infection is most prevalent in Latin America, sub-Saharan Africa, and South and Southeast Asia, including India—an endemic country for T. solium—increasing international migration has extended its relevance to non-endemic settings, where cases are increasingly diagnosed among migrant populations (5).

The clinical spectrum of NCC is highly pleomorphic and depends on the number, stage, and anatomical location of the cysts. Lesions are broadly categorized into viable (vesicular or colloidal) and calcified stages. Viable cysts are associated with active inflammation and may provoke seizures or other focal neurological deficits, whereas calcified lesions represent the terminal stage of cyst degeneration. Although calcified cysts no longer contain viable parasites, they may remain epileptogenic due to perilesional gliosis or intermittent inflammatory reactivation. Brain calcifications are commonly identified in individuals from endemic areas, and population-based studies suggest that 5–25% of asymptomatic individuals in such regions may harbor calcified parenchymal lesions. Seizures are the most frequent manifestation of NCC, and the condition is recognized as one of the leading causes of adult-onset epilepsy in endemic areas (6).

Beyond seizures, there is growing recognition that NCC may be associated with a broad range of psychiatric and neurocognitive symptoms, including depression, anxiety, cognitive impairment, and, in rarer instances, psychosis (7, 8). Psychiatric manifestations have been particularly linked to parenchymal lesions involving the frontal and temporal lobes, implicating both structural disruption and neuroinflammatory cascades as potential mechanisms (9). The neuropsychiatric consequences of NCC are thought to arise from overlapping pathways: degenerating cysts may trigger inflammatory responses, edema, and gliosis, potentially interfering with fronto-temporal and limbic circuits responsible for cognition, mood, and perception (10). Additionally, NCC is a well-established epileptogenic disorder, and recurrent seizures may predispose to postictal or interictal psychosis (8). Psychosocial determinants—including stigma, social isolation, and reduced quality of life—may further compound the psychiatric burden (7). The coexistence of structural, inflammatory, epileptogenic, and psychosocial factors complicates attribution of psychiatric symptoms to a single etiological pathway and poses diagnostic and therapeutic challenges.

Psychosis, while less common than affective or cognitive symptoms, represents one of the most clinically significant and diagnostically challenging neuropsychiatric outcomes of NCC. Case reports have described hallucinations, delusions, paranoia, and disorganized thought processes in patients with radiologically confirmed NCC, often in association with epilepsy (11). However, systematic studies quantifying the prevalence of psychosis in NCC remain limited, and robust longitudinal data linking lesion characteristics, inflammatory activity, and psychiatric outcomes are scarce (7). This knowledge gap underscores the need for detailed case-based reports to enhance clinical understanding, particularly in non-endemic settings.

Although NCC is classically endemic to low- and middle-income countries, cases are increasingly identified in high-income regions as a consequence of globalization and workforce mobility. Dubai, a cosmopolitan hub with a large expatriate population from endemic countries such as India, represents one such setting where NCC has emerging clinical relevance. The present case, diagnosed and managed at Rashid Hospital in Dubai, illustrates this evolving epidemiological landscape and highlights the importance of maintaining clinical vigilance for organic contributors to acute neuropsychiatric presentations in migrant populations. Furthermore, the complexity of this presentation required collaborative management between neurology and psychiatry teams, underscoring the necessity of multidisciplinary care in addressing the intertwined neurological and psychiatric dimensions of NCC.

In this context, we report the case of a young adult male from an endemic region who presented with acute psychosis and seizure history in whom neuroimaging revealed a calcified frontal NCC lesion with perilesional edema. This case highlights the diagnostic complexity of distinguishing primary psychiatric disorders from secondary psychosis potentially associated with underlying neurological pathology and provides an opportunity to examine current mechanistic hypotheses regarding psychosis in parasitic CNS infections.

## 2 Case presentation

The patient is a 23-year-old Indian male who presented to the emergency department in the United Arab Emirates (UAE) on September 14, 2025, with two days of abnormal behavior, confusion, and agitation.

The patient holds a bachelor’s degree in business administration and had recently relocated from India to the UAE for administrative employment. Although his work was clerical in nature, he began residing in a labor camp about one month prior to admission, where poor living conditions led to significant sleep deprivation, averaging only two to three hours of sleep per night.

Two days before admission, the patient’s supervisor contacted a friend expressing concern about his sudden change in behavior. When his friends arrived, they found him unresponsive and seemingly “frozen,” giving irrelevant and incoherent answers to simple questions. They described him as restless, fearful, and unaware of his surroundings with racing thoughts and frequent muttering to himself. At times, he would suddenly play loud music without reason and repeatedly insist that he did not want to return to the labor camp. Believing exhaustion to be the cause, his friends moved him to a nearby hotel. However, despite the change in environment, he slept for only two to three hours and continued with the same behavior after waking up.

The following day, his cousin brought him to Abu Dhabi to stay temporarily. That evening, he was observed hiding behind a door, later entering the kitchen where his cousin’s wife was working, gesturing as though crying for help with a fearful expression and half-closed eyes. Later that night, he sent her a series of incoherent text messages, including *“Bottega,” “Veneta,” “Come car,” “My left eye open you come,”* and *“Come to room.”* He also exhibited frequent blinking, lip trembling, and purposeless repetitive hand movements. Upon subsequent interviews, he recalled little of these events.

After a brief period of sleep, he appeared calm the next morning but became agitated again by midday. His cousin arranged a flight for him to return to India; however, at the airport, he refused to board, claiming that his cousin was the one traveling, and snatched his passport. He became irritable and combative, prompting airport staff to deny him boarding. He was subsequently brought to the emergency department for evaluation.

In the hospital, the patient was first assessed by the emergency medicine team after presenting with restlessness and abnormal behavior. He was observed to be pacing aimlessly, occasionally pointing at his cousin while mimicking a gun gesture, and remaining mute yet maintaining eye contact, with rhythmic movements of the right arm and leg and frequent blinking. He was administered diazepam by the emergency physician, after which he became calmer but continued to respond incoherently. Following this, the emergency team referred him to the psychiatry service for further evaluation.

During the psychiatric assessment, conducted with collateral history obtained from his cousin, his presentation remained largely unchanged as he was still mute and intermittently restless.

The patient’s psychiatric history included a similar episode nine months earlier while on a trip in India, characterized by aggression, suspiciousness, and disorganized speech lasting four days. he was admitted for one week and treated with oral sodium valproate extended release 500 mg daily, cariprazine 3 mg daily, amisulpride 200 mg daily, and clonazepam 0.25 mg daily, with full recovery. The treating psychiatrist advised him to avoid stress and travel; however, the patient discontinued the medications on his own shortly after recovery.

He also had two generalized tonic–clonic seizures. The first occurred nine months earlier and was poorly recalled. The most recent occurred three weeks prior to the current presentation as he was residing in the labor camp following a febrile illness. He recalled going to bed with fever and waking on the floor confused and fatigued. Witnesses described whole-body jerking lasting 2–3 minutes, upward eye-rolling, and frothy oral secretions, followed by transient confusion. No medical attention was sought at that time. There was no family history of psychiatric or neurological illness. He reported past recreational drug use (marijuana, LSD, crystal methamphetamine) two years prior, which led to a brief admission for an acute substance-induced psychotic episode, but he denied any substance use since that time.

On presentation, vital signs were stable (BP 134/85 mmHg, HR 70 bpm, RR 18/min, T 36.9 °C, SpO_2_ 98%). He appeared alert but poorly responsive, with mutism, staring, and psychomotor retardation, and had no focal neurological deficits. Mental status examination revealed a constricted affect, soft, hesitant speech, incoherent thought processes, and persecutory delusions accompanied by auditory hallucinations described as voices warning him not to trust others. Insight and judgment were poor.

**Figure 1:**
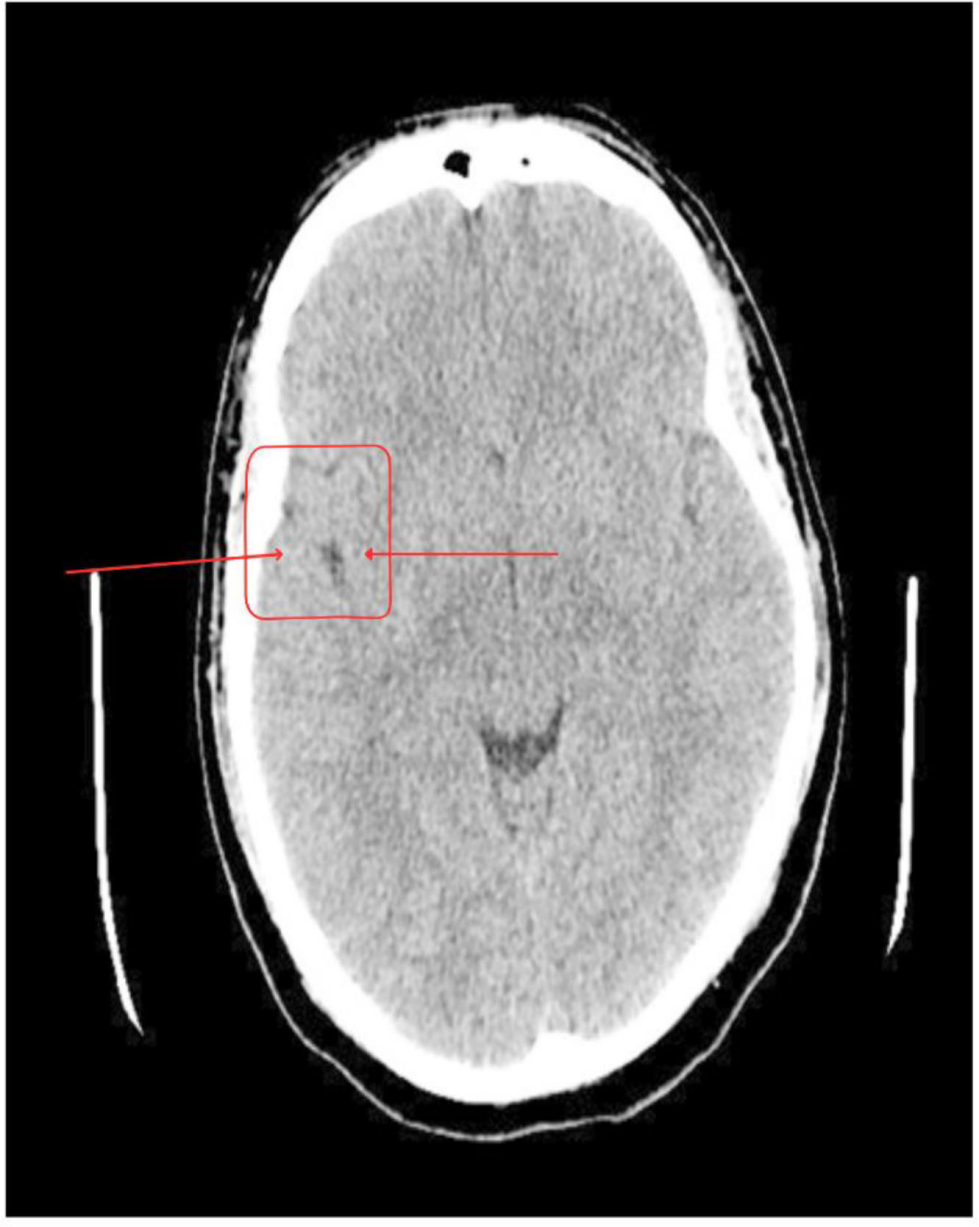
CT Brain (14 September 2025): A 3 mm cortical calcification with perilesional edema in the inferior posterior left frontal lobe, consistent with calcified neurocysticercosis.

Routine laboratory investigations were largely unremarkable except for mild lymphocytosis (3.1 × 10³/μL) and elevated creatine phosphokinase (263 U/L). Electrolytes and liver and renal function tests were normal. As part of the routine workup for altered mental status, a CT Brain (**Figure 1**) was performed on September 14, 2025, showing a 3 mm cortical calcification with perilesional edema in the inferior posterior left frontal lobe, consistent with calcified parenchymal neurocysticercosis. EEG was normal. Based on these findings, the patient was admitted under the Neurology service for further management, with the Psychiatry team continuing co-management of his psychotic and behavioral symptoms. On September 15, an MRI Brain with contrast was attempted but cancelled after the patient became acutely agitated, disoriented and attempted to leave the imaging suite. He was safely returned to the ward and later had no recollection of the event.

After admission, the patient was started on albendazole 400 mg twice daily for 2 weeks, dexamethasone 2 mg twice daily for 1 week, lamotrigine 25 mg twice daily titrated to 50 mg, risperidone 2 mg daily, and lorazepam 2 mg twice daily for catatonic features. Supportive care included sleep regulation, DVT and GI prophylaxis, and close observation.

Over the following four days, the patient’s condition improved markedly. By September 18, he was oriented, coherent, and free of psychotic symptoms, with restored sleep and appetite. The psychiatry team was following the patient on a daily basis and interviewing him but he could not recall most of the acute episode. He was discharged on September 18, 2025 on the same regimen as his family wanted to follow up with his care back in his home country.

**Figure 2:**
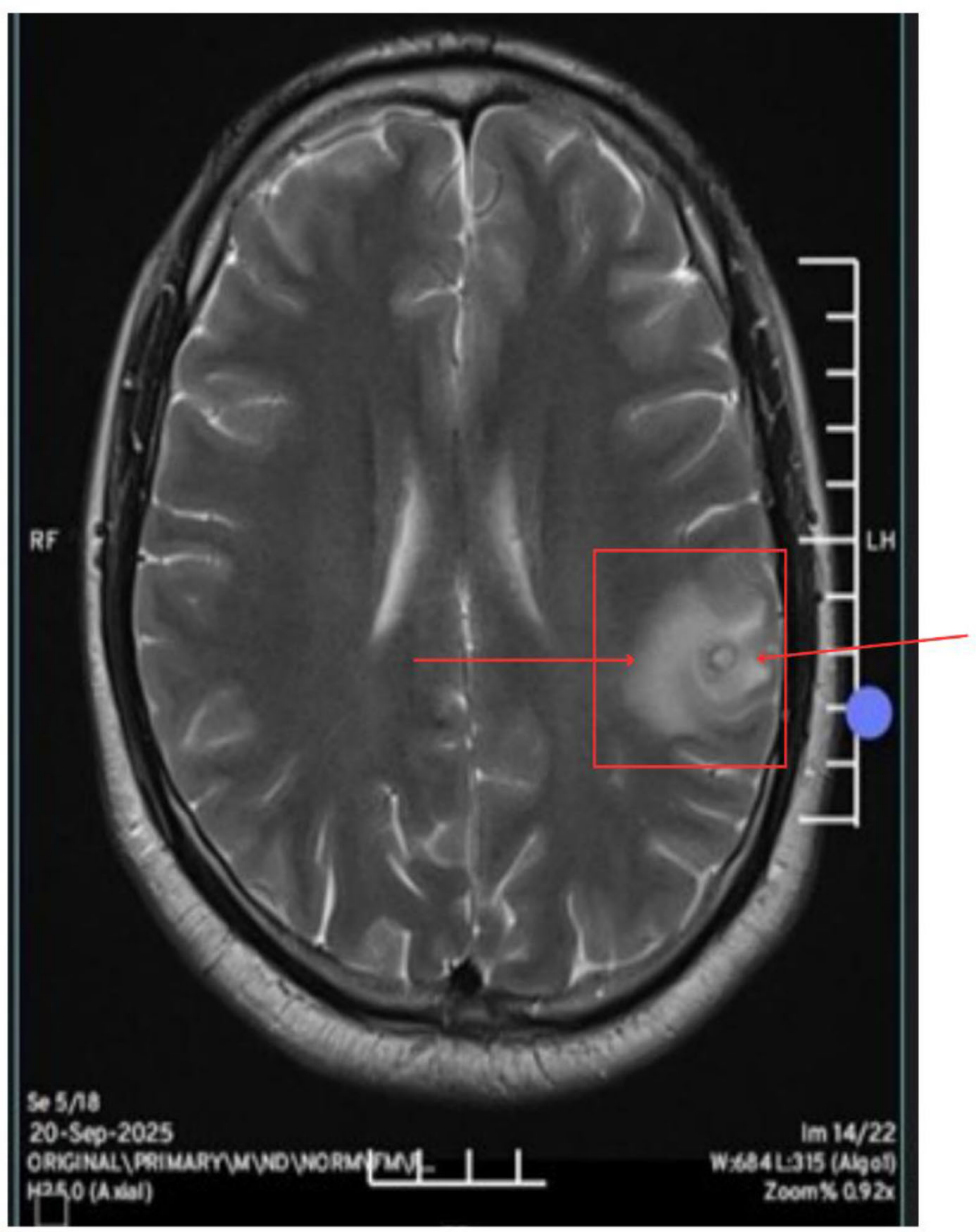
MRI Brain (20 September 2025, performed in India): Axial T2-weighted MRI showing a solitary ring-enhancing lesion with central calcification and mild surrounding edema in the left frontal region, suggestive of neurocysticercosis.

After discharge, the patient returned to India, where a follow-up MRI (**Figure 2**) performed on September 20, 2025, showed a solitary ring-enhancing lesion with central calcification and mild perilesional edema in the left frontal region, with features suggestive of neurocysticercosis.

**Figure 3:**
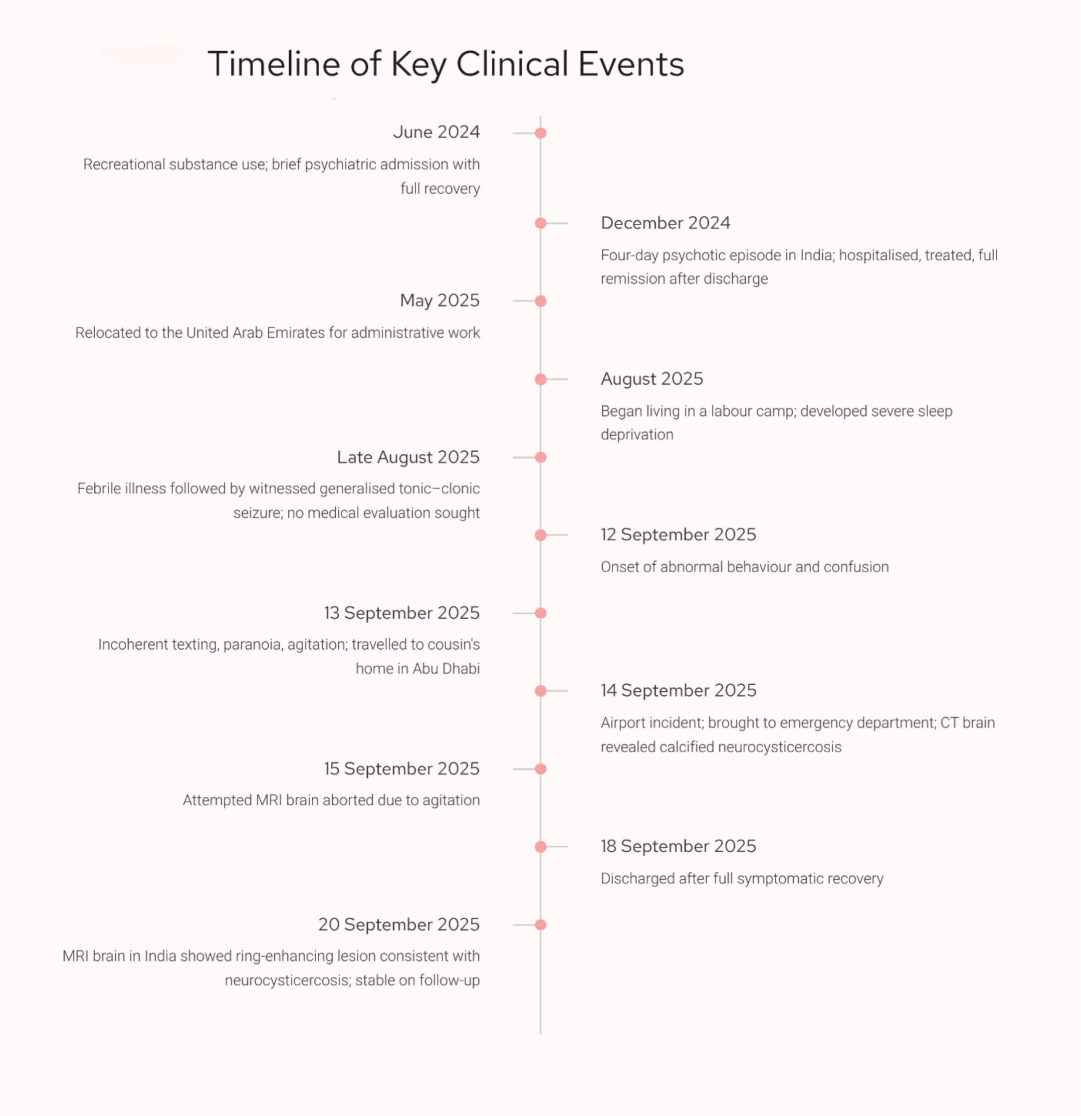
Timeline of key clinical events. Chronological summary of the patient’s major clinical events from June 2024 to September 2025, including prior psychiatric episodes, seizure history, onset of behavioural changes, emergency department presentation with neurocysticercosis, hospital management, and post-discharge follow-up imaging.

He is currently on lacosamide 50 mg daily and risperidone 2 mg daily, prescribed by his treating neurologist and psychiatrist in India. He remains under regular follow-up with both specialists, with reassessment scheduled within the next two months. To date, there have been no reported relapses of seizures, psychosis, or behavioral disturbances. The sequence of clinical events from the initial seizure episodes through hospital admission, treatment, and post-discharge follow-up is summarized in **Figure 3**.

## 3 Discussion

Neurocysticercosis (NCC) represents an important yet often underrecognized contributor to secondary psychiatric syndromes, capable of producing a broad spectrum of neuropsychiatric manifestations beyond its well-established epileptic presentations. The present case describes an acute psychotic episode occurring in a young adult with a calcified parenchymal NCC lesion and prior seizure history, managed through a multidisciplinary approach involving neurology and psychiatry. This case highlights the diagnostic and therapeutic challenges of distinguishing primary psychotic disorders from psychosis potentially associated with underlying neurological pathology in non-endemic, multicultural settings such as the United Arab Emirates.

Neurocysticercosis (NCC) manifests a pleomorphic clinical spectrum, determined by the number, viability, and neuroanatomical location of the parasitic cysts. Although seizures represent the most prevalent neurological manifestation and a leading etiology of acquired epilepsy in endemic regions (6), the neuropsychiatric burden of NCC is substantial. This includes affective and cognitive symptoms, with psychosis constituting a less frequent but clinically significant presentation that may emerge during both active and calcified stages, sometimes independent of overt neurological signs (7, 10). The index case illustrates this potential association, presenting with an acute psychotic episode characterized by agitation, mutism, paranoid delusions, and formal thought disorder. Neuroimaging localized a solitary calcified cyst with perilesional edema in the left inferior posterior frontal lobe. Involvement of this region, which encompasses Broca’s area and prefrontal circuits integral to executive control, social cognition, and language (9), provides a plausible neuroanatomical substrate for the observed symptoms, including disorganized thought (incoherence), avolition (mutism), and behavioral dysregulation (agitation).

**Figure 4:**
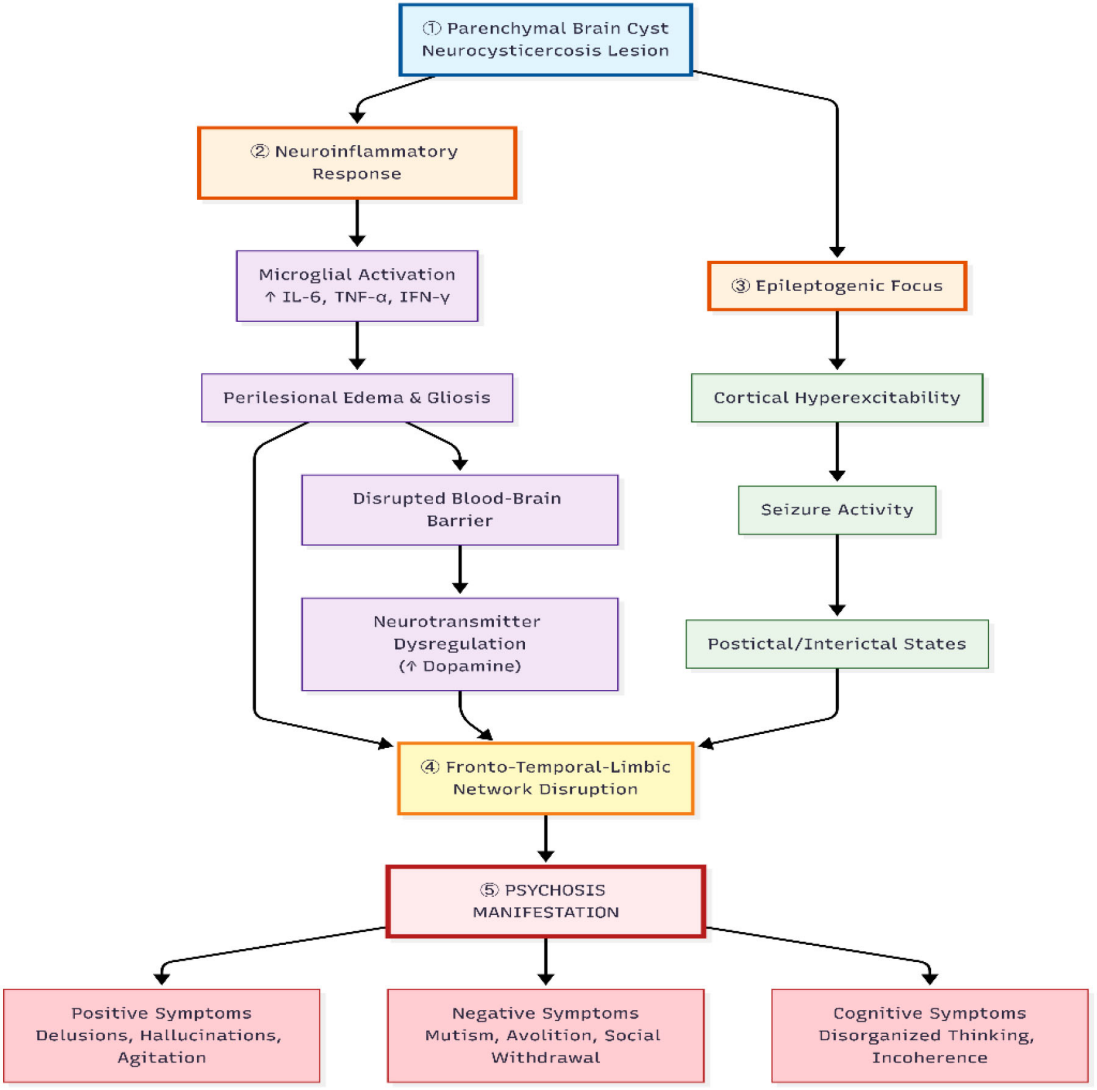
Proposed multifactorial mechanisms of psychosis in neurocysticercosis. The diagram illustrates how a parenchymal brain cyst (1) initiates a cascade of effects. The degenerating cyst triggers a local neuroinflammatory response (2) involving microglial activation and pro-inflammatory cytokines, leading to perilesional edema and gliosis. Concurrently, the cyst and inflammation act as an epileptogenic focus (3), predisposing to seizures. Both inflammation and seizure activity disrupt vulnerable neurocircuitry, particularly in the frontal and temporal lobes (4). This disruption of fronto-limbic circuits, potentially involving dopaminergic dysregulation, ultimately manifests as the positive and negative symptoms of psychosis (5).

The pathogenesis of NCC-related psychosis is likely multifactorial, involving a confluence of inflammatory, epileptogenic, and structural mechanisms that may disrupt cortico-subcortical circuitry. A central mechanism involves the host’s inflammatory response to degenerating cysticerci, characterized by microglial activation and the release of pro-inflammatory cytokines (e.g., IL-6, TNF-α, IFN-γ). This neuroinflammatory milieu can alter blood-brain barrier permeability, modulate neurotransmission—particularly within dopaminergic pathways—and promote cortical hyperexcitability (10). Concurrently, perilesional gliosis and edema may create an epileptogenic focus, predisposing to seizures and associated postictal or interictal psychotic states (8). As illustrated in **Figure 4**, these processes are not mutually exclusive but may interact synergistically, potentially contributing to disruption of fronto-temporal-limbic networks implicated in psychosis.

Differentiating secondary psychosis potentially associated with NCC from primary psychiatric disorders remains clinically demanding. Abrupt onset, fluctuating course, and the coexistence of neurological features such as seizures or focal deficits may favor an organic etiology (8). In this patient, the temporal association between prior seizures, imaging findings, and the acute psychotic episode supports consideration of a secondary mechanism. Moreover, the rapid clinical improvement observed following combined antiparasitic and corticosteroid therapy is consistent with the possibility of inflammation-related symptom exacerbation, although causality cannot be definitively established. The patient’s history of a nearly identical episode nine months prior, which also resolved with a similar treatment approach, may suggest a recurrent neuropsychiatric pattern temporally associated with NCC. However, while past substance use and significant psychosocial stressors (sleep deprivation, relocation) were present, these factors likely interacted with the underlying structural brain lesion rather than representing mutually exclusive explanations. Given the multifactorial nature of such presentations, cautious interpretation remains warranted. Such cases emphasize the importance of integrated neurological–psychiatric assessment and maintaining diagnostic vigilance in atypical psychotic presentations.

Management of NCC-associated psychiatric symptoms requires a tailored, multidisciplinary strategy. In this case, combined antiparasitic therapy (albendazole), corticosteroids, antiepileptic medication (lamotrigine), and antipsychotic therapy (risperidone) were followed by rapid symptom resolution. Because multiple treatments were initiated concurrently, the relative contribution of each intervention cannot be clearly delineated. Prior studies suggest that psychiatric manifestations may improve as cystic lesions stabilize and neuroinflammation decreases (1, 2). Psychiatric stabilization may facilitate adherence to neurological treatment, while seizure control may reduce the risk of psychosis recurrence. In endemic settings, early diagnosis and comprehensive management remain important for reducing both neurological and psychiatric morbidity.

The current case also underscores the global relevance of NCC in the context of migration and travel. Although traditionally confined to low- and middle-income countries, NCC is increasingly diagnosed in high-income regions due to population movement. Clinicians in non-endemic settings should therefore remain alert to NCC as a potential contributor to new-onset psychosis, particularly among individuals from endemic regions or those with seizure history. The case also highlights the importance of multidisciplinary coordination among neurology, psychiatry, and infectious disease teams in achieving favorable outcomes.

From a neuropsychiatric perspective, this case supports the conceptualization of NCC as a condition situated at the intersection of neurology, psychiatry, and infectious disease. It contributes to the growing body of evidence linking neuroinflammation, disrupted cortical connectivity, and immune-mediated processes to psychotic symptoms. Future research integrating neuroimaging, immunological profiling, and longitudinal follow-up is warranted to better delineate the temporal relationships between parasitic infection, inflammation, and psychiatric symptomatology.

Clinicians should maintain a high index of suspicion for NCC in patients presenting with acute psychosis, particularly when accompanied by seizure history or imaging abnormalities. Neuroimaging should be considered an essential component of the diagnostic workup for atypical or treatment-resistant psychosis. Early identification and integrated management can substantially improve clinical outcomes.

## 4 Conclusion

This neuropsychiatric case illustrates acute psychosis occurring in the context of calcified neurocysticercosis, with potential contributions from frontal lobe involvement, neuroinflammatory processes, and seizure-related network disruption. Clinical improvement was achieved through multidisciplinary collaboration involving neurology, psychiatry, and infectious disease teams, enabling comprehensive management addressing parasitic, inflammatory, epileptogenic, and psychiatric components. While the favorable treatment response supports a possible organic contribution, causal attribution remains multifactorial and should be interpreted cautiously. These findings highlight the importance of considering neuroimaging in first-episode or atypical psychosis evaluations, particularly among individuals from epidemiological risk populations. Furthermore, this case underscores the growing clinical relevance of NCC in non-endemic regions within the context of global migration. Future investigations integrating serial neuroimaging and immunological profiling may help clarify underlying neuropsychiatric mechanisms. Ultimately, this report reinforces the value of multidisciplinary neuropsychiatric assessment in optimizing diagnosis and management of complex secondary psychosis presentations.

## 5 Patient perspective

After recovery, the patient reported limited recollection of the acute episode but described the experience as distressing and confusing, particularly due to the sudden loss of control over his thoughts and behavior. He expressed relief after understanding that his symptoms had an identifiable and treatable neurological cause and reported reassurance from the rapid improvement following treatment. The patient emphasized the importance of coordinated neurological and psychiatric care, acknowledged the need for adherence to ongoing treatment and follow-up, and expressed hope to maintain long-term stability and resume normal daily activities.

## 6 Clinical significance

- Neurocysticercosis (NCC) should be recognized as a potential cause of acute psychosis, particularly in individuals from endemic regions or migrant populations, even when overt neurological signs are minimal or absent.
- Frontal lobe involvement and neuroinflammatory processes may precipitate psychosis through disruption of fronto-limbic circuits, dopaminergic dysregulation, and seizure-related network instability.
- Neuroimaging is essential in the evaluation of first-episode or atypical psychosis, as it can identify underlying organic etiologies that may otherwise be misclassified as primary psychiatric disorders.
- Multidisciplinary management incorporating antiparasitic, anti-inflammatory, antiepileptic, and antipsychotic therapies can result in complete symptom remission and reduce the risk of neurological and psychiatric relapse.
- Heightened clinician awareness in non-endemic settings is increasingly important as global migration expands the epidemiological footprint of NCC, reinforcing the need for integrated neuropsychiatric diagnostic and treatment approaches.

## Data Availability

The original contributions presented in the study are included in the article/supplementary material. Further inquiries can be directed to the corresponding author.
